# Selecting high-dimensional mixed graphical models using minimal AIC or BIC forests

**DOI:** 10.1186/1471-2105-11-18

**Published:** 2010-01-11

**Authors:** David Edwards, Gabriel CG de Abreu, Rodrigo Labouriau

**Affiliations:** 1 Institute of Genetics and Biotechnology, Faculty of Agricultural Sciences, Aarhus University, Aarhus, Denmark

## Abstract

**Background:**

Chow and Liu showed that the maximum likelihood tree for multivariate discrete distributions may be found using a maximum weight spanning tree algorithm, for example Kruskal's algorithm. The efficiency of the algorithm makes it tractable for high-dimensional problems.

**Results:**

We extend Chow and Liu's approach in two ways: first, to find the forest optimizing a penalized likelihood criterion, for example AIC or BIC, and second, to handle data with both discrete and Gaussian variables. We apply the approach to three datasets: two from gene expression studies and the third from a genetics of gene expression study. The minimal BIC forest supplements a conventional analysis of differential expression by providing a tentative network for the differentially expressed genes. In the genetics of gene expression context the method identifies a network approximating the joint distribution of the DNA markers and the gene expression levels.

**Conclusions:**

The approach is generally useful as a preliminary step towards understanding the overall dependence structure of high-dimensional discrete and/or continuous data. Trees and forests are unrealistically simple models for biological systems, but can provide useful insights. Uses include the following: identification of distinct connected components, which can be analysed separately (dimension reduction); identification of neighbourhoods for more detailed analyses; as initial models for search algorithms with a larger search space, for example decomposable models or Bayesian networks; and identification of interesting features, such as hub nodes.

## Background

Recent years have seen intense interest in representing complex biological systems as networks, and a new research discipline, network biology, has arisen. In particular, Markov networks and Bayesian networks have been applied in many domains [1-3]. The former are based on undirected graphs, and the latter on DAGs (directed acyclic graphs). A key challenge in deriving such networks from the high-dimensional data typical of the genomics era is computational efficiency: model selection algorithms that perform well for small or moderate dimensions may be intractable for high dimensions. The approach of Chow and Liu [4], which predates much of the development of probabilistic graphical models, is particularly efficient, being quadratic in the number of variables.

### The Chow-Liu algorithm

Suppose that we have a dataset with *N *observations of *p *discrete random variables *X *= (*X*_*v*_)_*v*∈Δ_. We call the possible values a discrete variable may take its *levels*, and label these 1,...|*X*_*v*_|, so that |*X*_*v*_| is the number of levels of *X*_*v*_. We write a generic observation (or *cell*) as *x *= (*x*_1_,..., *x*_*p*_), and the set of possible cells as *χ*. We assume that the observations are independent and are interested in modelling the probabilities *p*(*x*) = Pr(*X *= *x*) for *x *∈ *χ*.

Suppose also that the cell probabilities factorize according to a tree, that is, a connected acyclic graph, written  = (*X*, *E*) where *X *is the vertex set and *E *the set of edges. That is to say, the cell probabilities can be written *p*(*x*) = ∏_*e*∈*E*_*g*_*e*_(*x*) for functions *g*_*e*_(*x*) that only depend on the variables in *e*. So when *e *= (*X*_*u*_, *X*_*v*_), *g*_*e*_(*x*) is a function of *x*_*u *_and *x*_*v *_only. Chow and Liu [4] showed that the cell probabilities take the form(1)

where *d*_*v *_is the degree of *v*, that is, the number of edges incident to *v*. Hence up to a constant the maximized log-likelihood is ∑_(*u*, *v*)∈*E*_*I*_*u*, *v*_, where *I*_*u*, *v *_is given by

*n*(*x*_*u*_, *x*_*v*_) being the number of observations with *X*_*u *_= *x*_*u *_and *X*_*v *_= *x*_*v*_. The quantity *I*_*u*, *v *_is called the *mutual information*. It follows that if we use the *I*_*u*, *v *_as edge weights on the complete graph with vertex set *X*, and apply a maximum spanning tree algorithm, we obtain the maximum likelihood tree.

In statistical terms, *I*_*u*, *v *_is one half of the usual likelihood ratio test statistic for marginal independence of *X*_*u *_and *X*_*v*_, that is *G*^2 ^= -2 ln *Q *= 2*I*_*u*, *v*_, calculated using the table of counts {*n*(*x*_*u*_, *x*_*v*_)} formed by cross-tabulating *X*_*u *_and *X*_*v*_. Under marginal independence *G*^2 ^has an asymptotic  distribution, where *k *= (|*X*_*u*_| - 1)(|*X*_*v*_| - 1). The degrees of freedom *k *is the number of additional free parameters required under the alternative hypothesis, compared with the null hypothesis.

A very similar exposition can be given for multivariate Gaussian data: here the sample mutual information is

where  is the sample correlation between *X*_*u *_and *X*_*v*_. As before the likelihood ratio test statistic *G*^2 ^= -2 ln *Q *= 2*I*_*u*, *v*_. Under marginal independence *G*^2 ^has a  distribution.

Algorithms to find the maximum weight spanning tree of a arbitrary undirected connected graph  with positive edge weights have been studied thoroughly. The following simple and efficient algorithm is due to Kruskal [5]. Starting with the null graph, repeat this step: among the edges not yet chosen, add the edge with the largest weight that does not form a cycle with the ones already chosen. When *p *- 1 edges have been added, the maximum weight spanning tree of  has been found. The algorithm can be implemented to run in *O*(*p*^2 ^ln *p*) time.

As mentioned above,  is here taken to be the complete graph on *X *with edge weights given by {*I*_*u*, *v*_}_*u*, *v*∈*X*_. In practice the task of calculating these *p*(*p *- 1)/2 edge weights dominates the time usage, so the complexity of the Chow-Liu algorithm may be taken to be *O*(*p*^2^). Methods to improve computational efficiency have been described [6,7].

Chow and Liu's approach has been extended to more general classes of graphs than trees: to thin junction trees [8]; to polytrees [9]; to bounded tree-width networks [10], and to mixtures of trees [11]. The approach has also been extended to tree-based models for Gaussian processes [12] and discrete-valued time series [13]. The consistency of the algorithm has been shown [14].

## Results and Discussion

### Extension to minimal AIC/BIC forests

A disadvantage with selecting a tree based on maximum likelihood is that it will always include the maximum number of edges, irrespective of whether the data support this or not. It is desirable to take account of the number of model parameters in some fashion. In the machine learning literature it is customary to penalize the likelihood using the minimum description length principle [15], whereas in the statistical literature the use of information criteria is well-established, particularly AIC (the Akaike information criterion [16]) and BIC (the Bayesian information criterion [17]). The former is defined as -2 ln *L *+ 2*r*, where *L *is the maximized likelihood under the model and *r *is the number of parameters in the model, and the latter as -2 ln *L *+ ln(*N*)*r*. Discussions of the relative merits of these criteria are available [18] and need not be repeated here.

First, suppose that Kruskal's algorithm is applied using penalized mutual information quantities  = *I*_*u*, *v *_- *k*_*u*, *v *_or  = *I*_*u*, *v *_- ln(*N*)*k*_*u*, *v*_/2, where *k*_*u*, *v *_is the degrees of freedom associated with *I*_*u*, *v*_, as described above. Then it is easily seen that the tree with the minimum AIC or BIC is obtained. Note that for Gaussian data this will be identical to the maximum likelihood tree, since all edges have the same degrees of freedom. For discrete data with varying numbers of levels, the maximum likelihood tree and the minimal AIC/BIC tree will generally differ.

Second, given a graph  = (*V*, *E*) with both positive and negative edge weights, consider the problem of finding the maximum weight forest, that is, the acyclic subgraph on vertex set *V *with maximum weight. Let  be the graph derived from  by omitting all edges with negative weights. For any forest with vertex set *V*, removing all edges with negative weights would increase the total weight and not introduce any cycles. It follows that we can construct the maximum weight forest by finding the maximum weight spanning tree for each connected component of . We can do this simply by applying Kruskal's algorithm to : it is not necessary to find the connected components explicitly.

So it is easy to find the minimal AIC or BIC forest by using penalized mutual information quantities as weights. This approach is attractive with high-dimensional data, since if the selected forest does consist of multiple connected components these may then be analyzed separately -- allowing a dimension reduction. We show below that the connected components of the minimal AIC/BIC forest are also connected components of the minimal AIC/BIC decomposable model, providing further justification for this procedure.

That using penalized likelihood with the Chow-Liu algorithm leads to forests rather than trees appears to be known in the machine learning literature [19]; also, [20] finds the Bayesian MAP tree/forest in a similar way, but we have found no published references in the computational biology or statistics research literatures. We believe that it is a useful method that deserves to be far more widely known.

### A numerical illustration

Here we compare application of the algorithms to some simulated data involving three discrete random variables, *X*_*a*_, *X*_*b *_and *X*_*c *_with 2, 5, and 5 levels respectively, and whose joint distribution is given by

where Pr(*x*_*a*_) = (0.5, 0.5)',  and either (i)  or (ii) .

Note that *X*_*a *_and *X*_*b *_are strongly associated but there is weak or no association between *X*_*a *_and *X*_*c*_.

**Figure 1:**
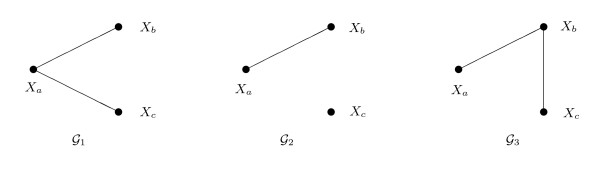
**Graphs connected with the simulations**. Data were simulated from , in case (i), and from , in case (ii). The third graph  is sometimes selected by the algorithms.

Figure 1 shows the corresponding independence graphs: in case (i), , and in case (ii), . A random dataset with 500 observations was drawn from each of the joint distributions and the algorithms applied. This was repeated 1000 times. The results are shown in Table 1.

**Table 1:** Simulation Results

	Case (i)			Case (ii)		
Algorithm						
ML tree	826	0	174	5	0	995
min AIC forest	1000	0	0	94	897	9
min BIC forest	995	5	0	0	1000	0

In case (i), the ML tree algorithm incorrectly identifies  about 17% of time; otherwise it correctly identifies . Penalizing with AIC or BIC increases the success frequencies to almost 100%. In case (ii) the true model  is a forest rather than a tree, so the ML tree algorithm cannot select it. Note that it almost always selects : since 2*I*_*b*, *c*_~  and 2*I*_*a*, *c *_~ , the former is almost always greater than the latter. Penalizing using AIC and BIC increases the success frequencies to 90% and 100%, respectively. For insight into the relative performance of AIC and BIC in this example, see [18].

### Extension to mixed discrete and Gaussian data

The second extension we consider is to data with both discrete and Gaussian variables. Our approach uses the class of undirected mixed graphical models [21-23]. Consider a data set with *N *observations of *p *discrete random variables *X *= (*X*_1_,... *X*_*p*_), and *q *continuous random variables *Y *= (*Y*_1_,... *Y*_*q*_). The models are based on the conditional Gaussian distribution, that is to say, the conditional distribution of *Y *given *X *= *x *is multivariate Gaussian with mean, and possibly also variance, depending on *x*. Models in which the variance depends on *x *are termed heterogenous, otherwise, they are called homogeneous.

**Figure 2:**
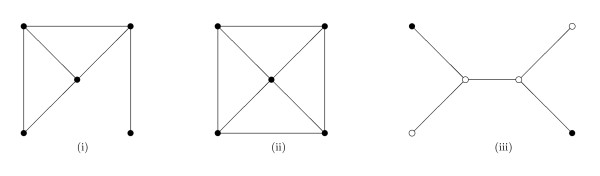
**Three undirected graphs**. Graph (i) is triangulated, that is, contains no chordless cycles of length four or greater. Graph (ii) is not triangulated, since it contains a chordless cycle of length four. Graph (iii) contains both discrete nodes (dots) and continuous nodes (circles). It is triangulated and contains a forbidden path.

Tree (or forest) dependence models can be defined as mixed graphical models whose independence graphs are trees (or forests). But since their likelihood functions do not in general factorize according to (2) the theory does not carry through directly. To obtain the analogous factorization, we restrict attention to those models that have explicit maximum likelihood estimates, the so-called strongly decomposable models [21,22,24]. These are easily characterized. A mixed graphical model is strongly decomposable if and only if it is triangulated (that is, contains no chordless cycles of length greater or equal to four) and contains no *forbidden paths *[22]. See Figure 2.

A forbidden path is a path between two non-adjacent discrete vertices passing through continuous vertices. Since trees and forests are acyclic, they are triangulated, and since they contain at most one path between any two vertices, we can simplify the criterion as follows: A tree or forest dependence model is strongly decomposable if and only if it contains no path between discrete vertices passing through continuous vertices. We call such a tree (or forest) an SD-tree (or SD-forest). In an SD-tree the discrete vertices induce a connected subgraph.

To apply the algorithm we need to derive the mutual information between a discrete variable *X*_*u *_and a continuous variable *Y*_*v*_. The marginal model is a simple ANOVA model (section 4.1.7 of [21]). Let *s*_0 _= ∑_*k*_(*y*^(*k*) ^- )^2^/*N*, and write the sample cell counts, means and variances as . In the homogeneous case, the mutual information is *I*_*u*, *v *_= *N *ln(*s*_0_/*s*)/2, where . There are *k *= |*X*_*u*_| - 1 degrees of freedom. In the heterogeneous case, the mutual information is *I*_*u*, *v *_= *N *ln(*s*_0_)/2 - , with *k *= 2(|*X*_*u*_| - 1) degrees of freedom. The expressions given here assume that all parameters are estimable: when this is not so, they need to be modified slightly, but we omit the details.

We also need to modify Kruskal's algorithm. As before an undirected graph  with positive weights is given. Starting with the null graph, we repeatedly add the edge with the largest weight that does not form a cycle or a forbidden path. It is shown below that this returns the maximum weight SD-forest.

### About the forbidden path restriction

**Figure 3:**
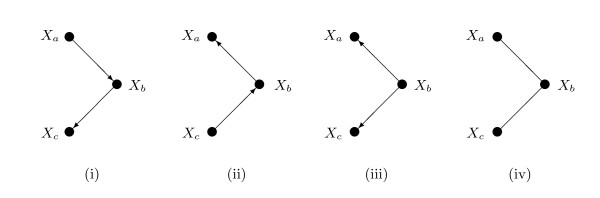
**Markov equivalence**. The first three graphs are DAGs, the fourth is undirected. All four graphs represent the same conditional independence relation: that *X*_*a *_and *X*_*c *_are conditionally independent given *X*_*b*_. They are called *Markov equivalent*.

We describe here a perspective on the forbidden path restriction that gives useful insight. Graphical models encode sets of conditional independence relations, and if two graphical models encode the same set of conditional independence relations they are termed *Markov equivalent *[25,26]. For example, each graph in Figure 3 represents the conditional independence of *X*_*a *_and *X*_*c *_given *X*_*b*_. Sample data from the joint distribution of *X*_*a*_, *X*_*b *_and *X*_*c *_supply information on which conditional independence relations hold and which do not, but cannot distinguish between the four graphs. To do this would require intervention in the system, for example by perturbing *X*_*a *_to see whether the distribution of *X*_*b *_is altered. For this reason algorithms to identify Bayesian networks from sample data [27,28] can only do this up to Markov equivalence.

**Figure 4:**
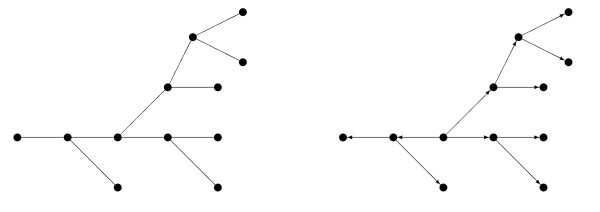
**A tree and a rooted tree**. Specifying a root generates a single-parent DAG.

The DAGs that are Markov equivalent to a given tree comprise a Markov equivalence class. As illustrated in Figure 4, they are easily found. Labelling a node (*X*_*r*_, say) as a root and orienting all edges away from the root, induces a single-parent DAG, that is, one in which all nodes have at most one parent. Any node can be chosen as root. Under such a DAG, the joint distribution factorizes into

where pa(*x*_*u*_) denotes the parents (here, parent) of *x*_*u *_in the DAG. Models corresponding to the DAG are constructed by specifying a marginal distribution Pr(*x*_*r*_) and a series of conditional models for Pr(*x*_*u*_|pa(*x*_*u*_)).

First consider the *pure *case, that is, when all variables are either discrete or continuous. In the discrete case, we can construct a model for the DAG by specifying a multinomial distribution for *X*_*r *_and arrays of transition probabilities for the conditional models. In the continuous case, *X*_*r *_is Gaussian and the conditional models are simple linear regressions. When *X*_*u *_and *X*_*v *_are both discrete or both continuous, the mutual information *I*_*u*, *v *_is symmetric, and is consistent with the conditional models for both Pr(*x*_*v*_|*x*_*u*_) and Pr(*x*_*u*_|*x*_*v*_). It follows that a DAG model in the Markov equivalence class is essentially a reparametrization of the tree model, and so has the same maximized likelihood and penalized likelihood scores. So in the pure case the algorithm identifies a Markov equivalence class of DAGs, just like other Bayesian network selection algorithms. Note that the search space is restricted to single-parent DAGs.

In the *mixed *case, however, the mutual information between a discrete *X*_*u *_and a continuous *X*_*v *_is asymmetric, and corresponds to an ANOVA-type conditional model for Pr(*x*_*v*_|*x*_*u*_) but not for Pr(*x*_*u*_|*x*_*v*_). So a DAG model in the Markov equivalence class is a reparametrization of the tree model only if the DAG contains no edges pointing from continuous to discrete nodes. If the tree has a forbidden path, no such DAG will exist: see for example Figure 2(iii). If the tree has no forbidden paths, then a DAG generated in the above way will have this property if and only if its root is discrete. So in the mixed case the algorithm identifies a subset of a Markov equivalence class of DAGs, those generated using discrete roots. That only a subset is identified is due to a limitation of the model apparatus, not to any evidence in the data. The limitation is unproblematic provided that the discrete variables are prior to the continuous variables.

All this has two broad implications. The first is that, when interpreted causally, the tree and forest models allow at most one determinant of each variable. The second is that the approach implicitly assumes that discrete variables are prior to continuous ones.

### A marginality property

In some cases the global optimality of the selected model holds under marginalization. The following result is shown below in the methods section. Suppose that  is the maximum likelihood tree (or minimal AIC or BIC forest) for a variable set *V *and let the connected components of  be *C*_1_,... *C*_*k*_, say. Then  (the marginal subgraph induced by *A *⊆ *V*) is the maximum likelihood tree (respectively, minimal AIC or BIC forest) for the variable set *A *provided that  is connected, for each component *C*_*i*_.

For example, consider a genetics of gene expression study involving a set of discrete DNA markers Δ and a set of continuous gene expression variables Γ. A central tenet is that DNA can affect gene expression but not vice versa. Suppose that the minimal AIC/BIC forest for *V *= (Δ, Γ) is . The forbidden path restriction implies that for each connected component *C*_*i *_of ,  is connected. Hence  is the minimal AIC/BIC forest for the discrete data alone. It follows that  can be regarded as a chain graph model [22] with two blocks, Δ and Γ, with Δ prior to Γ, consistent with the tenet.

### Some applications of the algorithm

We show the results of applying the algorithm to three datasets.

#### Study of leucine-responsive protein (Lrp) in *E. coli*

The first dataset stems from a previously reported gene expression study [29]. The stated purpose of this was to identify the network of genes that are differentially regulated by the global *E. coli *transcription factor, leucine-responsive regulatory protein (Lrp), during steady state growth in a glucose supplemented minimal salts medium. Lrp has been reported to affect the expression of approximately 236 genes [30]. Gene expression in two *E. coli *bacteria strains, labelled lrp+ and lrp-, were compared using eight Affymetrix ecoli chips. The lrp+ strain is the control or wild type, and the lrp- strain is the experimental type, with the Lrp gene knocked-out. Four chips were hybridized with RNA from the lrp+ strain, and four chips with RNA from the lrp- strain. The raw data were preprocessed using standard methods and the algorithm applied to the derived data. The dataset had *N *= 8 observations and 7313 variables, comprising 7312 continuous variables (the log-transformed gene expression values) and one discrete variable, strain.

Our implementation of the algorithm (see below) took about 2 minutes on a laptop running Windows XP to find the minimal BIC forest. This is too large to display here, so instead we examine an interesting subgraph.

**Figure 5:**
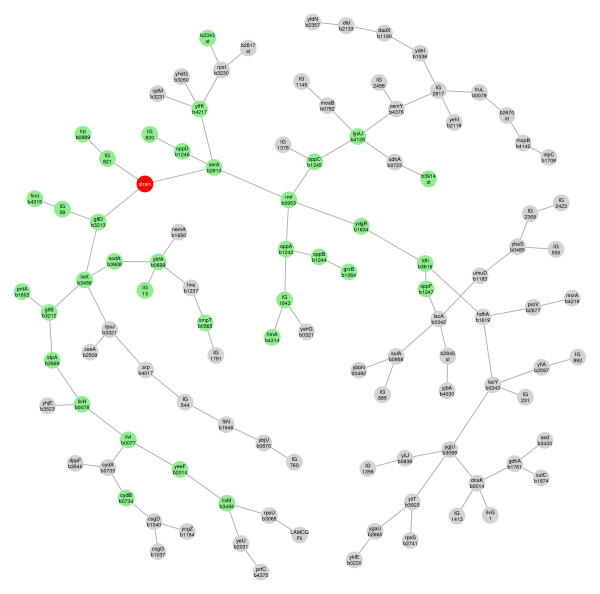
**The radius eight neighbourhood of **strain**in the minimal BIC forest for the E. coli data**. The class variable strain is shown as a red circle, and genes that are among the 40 top ranked in the limma analysis are shown as green circles.

Figure 5 shows the radius eight neighbourhood of strain, that is to say the subgraph of vertices whose path length from strain is less than or equal to 8. There are three variables adjacent to strain. The short arm links to the knockout gene itself via an intergenic region (IG) tRNA gene. This arm just reflects the marked downregulation of Lrp in the knockout strain. The other two arms suggest that Lrp targets just two genes, serA and gltD. It is instructive to compare Figure 4 with a conventional analysis of differential expression using the limma library [31]. If a false discovery rate of 0.2 is used, 40 genes are flagged as possibly differentially regulated. Although the two analysis approaches are very different -- limma is based on gene-by-gene hypothesis testing, and is concerned with the operating characteristics of this, while the present approach is based on approximating the joint distribution of the entire variable set -- the results are broadly consistent. Of the 40 genes identified by the limma analysis, 35 have a path length less or equal to 8 to strain in the minimum BIC forest, and so appear in Figure 5. The remaining 5 genes, however, are very distant from strain, with path lengths ranging from 59 to 81. This could suggest that their apparent regulation by Lrp is spurious.

The regulatory system of *E. coli *has been well-studied, and it is interesting to note that other studies confirm that serA and gltD are targets of Lrp [30,32]. Indeed, Lrp has many targets: 138 Lrp-binding sites have been identified [30], so it is certainly not true that Lrp only targets serA and gltD. We have not been able to find other reports that the five distant genes -- ndk, pnt, ptsG, nupG and atpG -- should be directly or indirectly regulated by Lrp.

The minimal BIC forest provides a provisional causal model for the effect of Lrp, and in this sense more directly addresses the stated goal of the study than a conventional analysis of differential expression. However, given the small number of observations in the study, it is clear that the network identification and any interpretations based on this are highly uncertain.

#### Gene expression profiling in breast cancer patients

The second dataset comes from another gene expression study [33], whose purpose was to compare the gene expression profiles in tumours taken from two groups of breast cancer patient, those with and those without a mutation in the p53 tumour suppression gene. A dataset containing a subset of the study data is supplied along with the R library gRbase. The dataset has *N *= 250 observations and 1001 variables, comprising 1000 continuous variables (the log-transformed gene expression values) and the class variable. There are 58 cases (with a p53 mutation) and 192 controls (without the mutation). The gene expression variables were filtered from a larger set, and all exhibit differential expression between the two groups. They have been standardized to zero mean and unit variance, but since the mixed graphical models used here are location and scale invariant, this does not affect the analysis.

**Figure 6:**
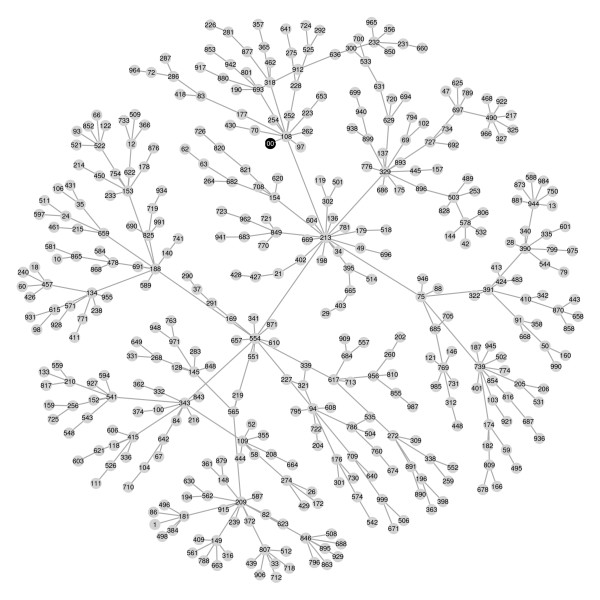
**The radius seven neighbourhood of the class variable in the minimal BIC forest for the breast cancer data**. The class variable is shown as a black circle.

The algorithm took about 18 seconds to find the minimal BIC forest. Figure 6 shows the radius seven neighbourhood of the class variable. The graph suggests that the effect of the p53 mutation on the gene expression profile is mediated by its effect on the expression of a gene with column number 108. This gene is CDC20, a gene involved in cell division. To examine this hypothesis more critically we could apply a richer class of models to this neighbourhood of genes, but that would take us outside the scope of this paper. Figure 6 also shows some apparent hub nodes, including 209 (GPR19), 329 (BUB1), 213 (CENPA), 554 (C10orf3) and 739 (CDCA5), that appear to play a key role in the system. See table 2 of [33] for further information on p53 - associated genes.

#### Genetics of gene expression using HapMap data

The third dataset comes from a large multinational project to study human genetic variation, the HapMap project http://www.hapmap.org/. The dataset concerns a sample of 90 Utah residents with northern and western European ancestry, the so-called CEU population, and contains information on genetic variants and gene expression values for this sample. The subjects are not unrelated (they comprise parent-sibling trios), but the analysis ignores this. The genetic variants are SNPs (single nucleotide polymorphisms). Datasets containing both genomic and gene expression data enable study of the the genetic basis for differences in gene expression. This dataset is supplied along with the R library GGtools.

**Figure 7:**
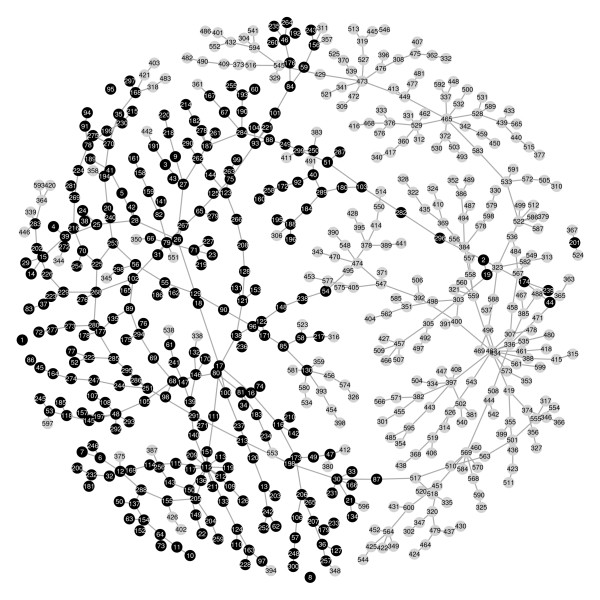
**The minimal BIC forest for the HapMap data**. There are five connected components: the main component has 594 nodes, there is one with three nodes and there are three isolated nodes.

For illustrative purposes, the first 300 polymorphic SNPs and 300 gene expression values are here used in the analysis. If non-polymorphic SNPs were included, they would appear as isolated vertices in the SD-forest, but it is more efficient to exclude them beforehand. As may be characteristic for SNP data, there are many ties in the mutual information quantities, so there may be multiple SD-forests with minimal BIC. The algorithm took about 2 seconds to find the one shown in Figure 7 below.

The main component of the SD-forest consists of a large connected block of SNPs, attached to most of the gene expression nodes via SNP number 87 at the bottom of the figure. There are also 30 or so gene expression nodes adjacent to the SNPs as singletons, and a component of nine gene expression variables connected to SNP number 54 in the centre of the graph. SNP number 130 is possibly a gene expression hotspot and there are several potential hub nodes among the gene expression values.

The SD-forest does not allow study of the joint effect of SNPs on gene expression values since, as we have seen, in trees and forests variables may have most one determinant. The minimal BIC forest obtained can be regarded as a special case of a chain graph model with two blocks, with the SNP data in the first block and transcript abundance data in the second block, as mentioned above. This framework would be well-suited for further analysis of the data, allowing study of the joint action of SNPs on gene expression values.

## Discussion

Deriving networks from high-dimensional data is a key challenge in many disciplines, and many different approaches have been proposed: for example, using approximation techniques [34] or low-order conditional independence tests [35,36]. One broad approach is to consider restricted classes of graphs, for example triangulated graphs [37], interval graphs [38] and others mentioned above, for which faster algorithms can be applied. The Chow-Liu algorithm falls into this class. Its utility is due to its remarkable computational efficiency, which reflects the simplicity of the graphs used. At the other end of the spectrum, it has been shown that selecting general Bayesian networks by maximizing a score function is NP-hard [39].

In this paper we have described some simple extensions to Chow and Liu's method that enable forests with minimal AIC or BIC to be found, and allow datasets with both discrete and Gaussian variables to be handled. In the previous section we demonstrated that useful insights into various high-dimensional datasets may be obtained by this method.

Trees and forests are too simple to be realistic models of complex biological systems. Nevertheless we believe that they can give a preliminary understanding of the overall dependence structure, and can be put to a number of practical uses.

Firstly, we can use the selected model as a start model in a search algorithm based on richer, but more computationally demanding, model classes. Since trees are triangulated, the class of (strongly) decomposable models is a natural choice for high-dimensional data. As described above, trees and forests represent Markov equivalence classes of DAGs, so the minimal AIC/BIC forest can also be used as start model in Bayesian network search procedures.

Secondly, we can regard properties of the selected model as proxies for corresponding properties of the true, underlying network. Properties that can be used in this way include connectivity, path length and degree. Provided we can assume that the data are generated by a joint undirected model, we can model the connected components of the selected forest separately. This may allow substantial dimension reduction. It is natural to use the selected forest to identify neighborhoods of interesting variables for more detailed analysis: in effect, this uses path length in the forest as a proxy for minimum path length in the unknown true network. Similarly, we can identify interesting features such as hub nodes -- nodes of high degree -- that may play a special role in the true network.

Recently there has been interest in *network motifs *-- patterns of interconnections between small numbers of nodes that occur significantly more often than could be expected by chance [40]. For a review of motif discovery algorithms, see [41]. Many of these motifs, such as the feed-forward or bi-parallel motifs, will not appear in trees due to the single-parent restriction discussed above. For this reason trees and forests appear to be too restrictive for motif discovery.

As pointed out by a referee, there are some similarities between the Chow-Liu algorithm and the ARACNE algorithm [42]. Like the Chow-Liu algorithm, this algorithm initially computes the mutual information quantities *I*_*u*, *v *_for all node pairs (although ARACNE uses the Gaussian kernel method of [43]). It forms an initial graph  by including all edges for which the *I*_*u*, *v *_exceeds a given threshold. The data-processing inequality states that if *X*_*u *_and *X*_*w *_are conditionally independent given *X*_*v*_, then *I*_*u*, *w *_< min(*I*_*u*, *v*_, *I*_*v*, *w*_). This is used to prune all complete triplets in , that is, all triplets *X*_*u*_, *X*_*v*_, *X*_*w *_with all three edges present in , by removing the edge with the least mutual information. Since the condition given in the data-processing inequality is sufficient but not necessary, that the inequality holds does not imply that the condition is true, and the authors acknowledge that the process may incorrectly remove edges.

Nevertheless the heuristic is reported to perform well when the true graph is a tree or is tree-like [42].

Although mixed graphical models have been studied for some time [21-23], their adoption by the machine learning community seems to have been limited. As illustrated above, some natural application areas include comparative microarray studies, to model the effect of an intervention or class variable on gene expression, and genetics of gene expression studies, involving both discrete DNA markers (SNPs) and continuous responses (gene expression values). In both cases the discrete variables are clearly prior to the continuous variables. The conditional Gaussian assumption is a distributional assumption that is not necessarily fulfilled for all continuous variables; but log-transformed gene expression values have been found to be approximately Gaussian, and this assumption provides the basis for conventional analyses of differential expression.

An attractive aspect of the algorithm is that it allows different measures of mutual information to be used -- for example, measures based on specific genetic models. However, we consider it a key advantage of the models described here that they are embedded in a broader class of models for more general dependence structures, which provides an inferential framework for systematic model diagnostics and development.

## Conclusion

The approach is generally useful as a preliminary step towards understanding the overall dependence structure of high-dimensional discrete and/or continuous data. Trees and forests are unrealistically simple models for biological systems, but can nevertheless provide useful insights. In microarray studies the method supplements lists of differentially regulated genes, by suggesting a possible network of interrelationsships between these. Other uses include the following: identification of distinct connected components, which can be analysed separately (dimension reduction); identification of neighbourhoods for more detailed analyses; as initial models for search algorithms with a larger search space, for example decomposable models or Bayesian networks; and identification of interesting features, such as hub nodes.

## Methods

### Modifying Kruskal's algorithm to find the maximum weight spanning SD-forest

We take as given an undirected graph  = (*V*, ) with positive edge weights, whose vertices are marked as either discrete and or continuous. We assume that the weights are distinct so that there is a unique spanning SD-forest with maximum weight. We consider the following modification of Kruskal's algorithm.

Starting with the null graph, repeatedly add the edge with the largest weight that does not form a cycle or a forbidden path. We claim that this finds the maximum weight SD-forest.

To prove this, let *T *= (*V*, *E*_*T*_) be the maximum weight spanning SD-forest, and let the edges chosen by the algorithm be *a*_1 _... *a*_*k*_. Let *A*_*i *_= (*V*, *E*_*i*_) be the SD-forest consisting of edges *a*_1 _... *a*_*i*_, so that *E*_*i *_= ∪_1≤*j*≤*i*_{*a*_*j*_}. Suppose that *T *≠ *A*_*k*_. Then either or both of (i) *E*_*k *_⊈ *E*_*T *_and (ii) *E*_*T *_⊈ *E*_*k *_must hold.

Suppose that (i) holds, and let *a*_*i *_be the first edge of *A*_*k *_which is not in *E*_*T*_. The addition of *a*_*i *_to *T *must result in a cycle or a forbidden path. Let *a*_*i *_= (*u*, *v*) and let the connected components (trees) of *T *containing *u *and *v *be *S*_*u *_and *S*_*v*_.

Suppose first that *S*_*u *_≠ *S*_*v*_. Addition of an edge between distinct components cannot create a cycle, but may create a forbidden path. Addition of an edge between discrete vertices cannot create a forbidden path, so one or both of *u *and *v *must be continuous. Suppose that *u *is discrete and *v *is continuous. Then (*V*, *E*_*T *_∪ *a*_*i*_) contains a unique forbidden path of the form *u*, *v*, *v*_1 _... *v*_*m*_, *w *for some *m *≥ 0 where *v*_1 _... *v*_*m *_are continuous and *w *is discrete. It is unique because the existence of two such paths would imply the existence in *S*_*v *_of a cycle (if the paths have the same *w*) or a forbidden path (if they have different *w*'s). Since *A*_*i *_is an SD-forest at least one edge in this path, say *e*, must be absent from *A*_*i*_. Then (*V*, *E*_*i*-1 _∪ *e*) is a SD-forest since it is contained in *T*. So the weight of *e *must be less than that of *a*_*i*_. Consider (*V*, *E*_*T*_\*e*). The removal of *e *from *S*_*v *_results in two subtrees, the one with *v *containing continuous vertices only. Hence (*V*, *E*_*T *_∪ *a*_*i*_\*e*) is an SD-forest. But the weight of (*V*, *E*_*T *_∪ *a*_*i*_\*e*) is greater than that of *T*, contradicting the definition of *T*. The proof when both *u *and *v *are continuous is similar.

Suppose now that *S*_*u *_= *S*_*v*_. Then (*V*, *E*_*T *_∪ *a*_*i*_) contains exactly one cycle, and may also contain a forbidden path. The cycle must contain *a*_*i *_and also some edge *e *which is not in *A*_*k*_. Then (*V*, *E*_*T *_∪ *a*_*i*_\*e*) is a forest. Suppose that (*V*, *E*_*T *_∪ *a*_*i*_) contains no forbidden path. Then (*V*, *E*_*T *_∪ *a*_*i*_\*e*) is an SD-forest. Since (*V*, *E*_*i*-1 _∪ *e*) is contained in *T*, it is an SD-forest, so the weight of *e *is less than that of *a*_*i*_. But then the weight of (*V*, *E*_*T *_∪ *a*_*i*_\*e*) is greater than that of *T*, contradicting the definition of *T*.

Suppose now that (*V*, *E*_*T *_∪ *a*_*i*_) contains a forbidden path, and let *a*_*i *_= (*u*, *v*). Suppose that *u *is discrete and *v *continuous. Then (*V*, *E*_*T *_∪ *a*_*i*_) contains a unique forbidden path of the form *u*, *v*, *v*_1 _... *v*_*m*_, *w *for some *m *≥ 0 where *v*_1 _... *v*_*m *_are continuous and *w *is discrete. Let *w*, *w*_1 _... *w*_*n*_, *u *for some *n *≥ 0 be the unique path in *S*_*u *_between *w *and *u*. Since *S*_*u *_is an SD-tree *w*_1 _... *w*_*m *_are discrete. Then the unique cycle in (*V*, *E*_*T *_∪ *a*_*i*_) takes the form *u*, *v*, *v*_1 _... *v*_*m*_, *w*, *w*_1 _... *w*_*n*_, *u*. Since *A*_*i *_is an SD-forest at least one edge in the path *u*, *v*, *v*_1 _... *v*_*m*_, *w*, say *e*, must be absent from *A*_*i*_. Removal of *e *from (*V*, *E*_*T *_∪ *a*_*i*_) breaks the cycle and the forbidden path, so (*V*, *E*_*T *_∪ *a*_*i*_\*e*) is an SD-forest. As before the weight of *e *is less than that of *a*_*i*_, so the weight of (*V*, *E*_*T *_∪ *a*_*i*_\*e*) is greater than that of *T*, contradicting the definition of *T*. The proof when both *u *and *v *are continuous is similar.

Hence *E*_*k *_⊆ *E*_*T*_.

Suppose now that (ii) holds. But any edge *e *∈ *E*_*T*_\*E*_*k *_would give rise to a cycle or a forbidden path if added to *E*_*k*_. Since *E*_*k *_⊆ *E*_*T *_this implies that *T *contains a cycle or forbidden path, contradicting its definition. It follows that *E*_*T *_⊆ *E*_*k *_and hence *T *= *A*_*k *_as required.

### Two theoretical properties of minimal AIC or BIC forests

In this section we prove the two theoretical properties of the selected models discussed above.

Firstly, suppose that we apply the algorithm to find the minimal AIC or BIC forest, say . Then the connected components of  are identical to the connected components of the minimal AIC/BIC strongly decomposable model. To see this, consider the connected components (that is, trees) of . Then any inter-component edge either corresponds to a negative penalized mutual information or would generate a forbidden path (since adding such an edge cannot form a cycle).

Suppose that we construct a global model * by using the strongly decomposable model with minimal AIC/BIC for each connected component of . It follows from decomposition properties of undirected graphical models [22] that adding an inter-component edge to * would result in the same change in AIC/BIC as when added to . Furthermore, if adding such an edge to  would generate a forbidden path it would do the same when added to *. So * is, at least locally, a minimal AIC/BIC strongly decomposable model.

Secondly, in some cases the global optimality of the selected model holds under marginalization. That is to say, if  is the maximum likelihood tree (or minimal AIC or BIC forest) for a variable set *V*, then for some variable subsets *A *⊆ *V*, the induced marginal subgraph of  on *A*, written , is the maximum likelihood tree (respectively, minimal AIC or BIC forest) for the variable set *A*. It is useful to characterize precisely the sets *A *for which this property holds in general.

Suppose initially that  is connected, that is, a tree. We claim that the property holds precisely for those sets *A *for which  is connected. Write  = (*A*, *E*_*A*_) and consider application of the algorithm to *A*, that is, to the subset of the (possibly penalized) mutual information quantities that pertain to *A*. Suppose that this generates the graph ℋ = (*A*, E*). We need to show that when the algorithm is applied to *V*, the inclusion of an edge between vertices in *A *cannot create a cycle or forbidden path involving edges not in *A*. If this occurs during the course of the algorithm, it will also occur when added to , so it is sufficient to consider . If  is connected then precisely one vertex in each connected component of  is adjacent to precisely one vertex of . So clearly the addition of an edge in *A *cannot create a cycle with edges not in *A*. Suppose it creates a forbidden path involving vertices not in *A*. This must link two discrete variables, say *u *and *v*, in distinct connected components of . Since  is an SD-tree, all vertices in the unique path between the two vertices in  must be discrete. This path must include the two vertices, say *w *and *x*, that are adjacent to a vertex in the connected components. If inclusion of an edge in *A *creates a forbidden path between *u *and *v*, then this must pass through *w *and *x*. But then the forbidden path lies in *A*, contrary to assumption. It follows that . Conversely, if  is not connected but  is, the inclusion of inter-component edges may give rise to cycles when the algorithm is applied to *V *but not when it is applied to *A*. Hence in general ℋ and  will differ.

When the minimal AIC or BIC variants of the algorithm are used,  may be a forest. Let the connected components of  be *C*_1_,... *C*_*k*_, say. Using a similar logic we obtain that  is the minimal AIC (or BIC) forest for the variable set *A *provided that  is connected, for each *i*.

## Availability

The analyses were performed using the R library gRapHD which we have made available to the R community via the CRAN repository (de Abreu GCG, Labouriau R, Edwards D: High-dimensional Graphical Model Search with gRapHD R package, submitted to J. Stat. Software).

## Authors' contributions

DE conceived the algorithm, performed the analyses and drafted the paper. GCGA carried out the programming effort. All authors contributed discussions to the theoretical development, and read and approved the final manuscript.
